# Evaluation of Vascular Patterns Using Contact Endoscopy and Narrow-Band Imaging (CE-NBI) for the Diagnosis of Vocal Fold Malignancy

**DOI:** 10.3390/cancers12010248

**Published:** 2020-01-20

**Authors:** Nikolaos Davaris, Anke Lux, Nazila Esmaeili, Alfredo Illanes, Axel Boese, Michael Friebe, Christoph Arens

**Affiliations:** 1 Department of Otorhinolaryngology, Head and Neck Surgery, Magdeburg University Hospital, 39120 Magdeburg, Germany; christoph.arens@med.ovgu.de; 2 Institute of Biometry and Medical Informatics, Otto-von-Guericke University, 39120 Magdeburg, Germany; Anke.Lux@med.ovgu.de; 3 Institute of Medical Technology, Otto-von-Guericke University Magdeburg, 39120 Magdeburg, Germany; nazila.esmaeili@ovgu.de (N.E.); alfredo.illanes@ovgu.de (A.I.); axel.boese@ovgu.de (A.B.); 4 Faculty of Medicine, Otto-von-Guericke-University, 39120 Magdeburg, Germany and IDTM GmbH, 45657 Recklinghausen, Germany; michael.friebe@ovgu.de

**Keywords:** laryngeal cancer, contact endoscopy, narrow-band imaging, vascular changes

## Abstract

The endoscopic detection of perpendicular vascular changes (PVC) of the vocal folds has been associated with vocal fold cancer, dysplastic lesions, and papillomatosis, according to a classification proposed by the European Laryngological Society (ELS). The combination of contact endoscopy with narrow-band imaging (NBI-CE) allows intraoperatively a highly contrasted, real-time visualization of vascular changes of the vocal folds. Aim of the present study was to determine the association of PVC to specific histological diagnoses, the level of interobserver agreement in the detection of PVC, and their diagnostic effectiveness in diagnosing laryngeal malignancy. The evaluation of our data confirmed the association of PVC to vocal fold cancer, dysplastic lesions, and papillomatosis. The level of agreement between the observers in the identification of PVC was moderate for the less-experienced observers and almost perfect for the experienced observers. The identification of PVC during NBI-CE proved to be a valuable indicator for diagnosing malignant and premalignant lesions.

## 1. Introduction

Changes in the morphology and three-dimensional spread of vocal fold vessels result from various functional, mechanical, or neoplastic stimuli and have been associated with the development of benign or malignant laryngeal pathologies, such as recurrent respiratory papillomatosis or laryngeal carcinoma [1,2,3]. Several classification systems have been proposed to describe and categorize subepithelial and epithelial vascular changes, while different image-enhancing technologies (electronic chromoendoscopy), such as narrow-band imaging (NBI), Storz professional image enhancement system (SPIES), or i-SCAN, have been used to ease their detection [4,5,6,7]. 

The Committee on Endoscopic Laryngeal Imaging of the European Laryngological Society (ELS) proposed in 2015 a simplified approach to the classification of vascular patterns of vocal folds [6]. It differentiates between two main categories: longitudinal and perpendicular vascular changes. Longitudinal vascular changes (LVC), including ectasia, meander, varicose, convolute, increased vessel number or branching, and change of direction, spread along the length and width of the vocal fold and can be observed in all kinds of benign or malignant laryngeal pathologies. Opposing that, perpendicular vascular changes (PVC), which represent intracapillary papillary capillary loops (IPCL), develop perpendicularly towards the mucosa as a result of neoplastic stimuli and have been associated with papillomatosis, premalignant, and malignant lesions. A further subdivision between wide or narrow-angled points in PVC can assist in the differentiation between (pre)malignant lesions and papillomatosis. Thus, the endoscopic detection of PVC has a diagnostic relevance and can be crucial for optimizing therapeutic decisions in the context of microlaryngoscopy and laryngeal surgery [6,8]. 

Contact endoscopy (CE), using methylene blue for tissue staining, was introduced to the laryngology in the 1990s, enabling the in vivo detection of cellular changes in order to diagnose malignancy [9]. The focus on endoscopic descriptions of vascular patterns in the last years revealed a new field of use. CE without tissue staining can be combined with image-enhancing technologies, such as NBI or SPIES, for the in vivo examination of superficial vessels with a magnification of 60× to 150× [5,10]. The use of NBI with its emitted wavelengths at 415 nm and 540 nm, instead of white light endoscopy (WLE), increases the vessel contrast as it highlights the superficial capillary network and submucosal vessels in deeper levels [11,12]. As a result, the combination of both modalities (NBI-CE) enables a detailed examination of vascular patterns and the on-site detection of minute PVC of vocal fold lesions during endoscopic laryngeal surgery [10]. Consequently, the interpretation of identified vascular changes using the newly proposed ELS classification while performing intraoperative endoscopy with NBI-CE has the potential to reduce the subjectivity in their evaluations, allowing the detection of PVC to be an independent endoscopic criterion in the diagnosis of laryngeal malignancy.

This study explores the value of the intraoperative detection of PVC using NBI-CE in terms of investigating its association to specific histological diagnoses, the level of interobserver agreement in the interpretation of vascular patterns using the ELS classification, and its diagnostic effectiveness in detecting laryngeal malignancy. 

## 2. Materials and Methods

Sixty-eight consecutive adult patients routinely planned for diagnostic microlaryngoscopy due to a lesion of the vocal folds have been included in this study between 1 January, 2017 and 31 August, 2018. A series of (four to five) images of unique vascular patterns in the mucosa of the target lesion were obtained using NBI-CE before performing an excisional biopsy or cordectomy of the vocal fold. 

The equipment used consisted of an Evis Exera III video system with a xenon light source, plus an integrated NBI filter (Olympus Medical Systems, Hamburg, Germany) and a rigid 30-degree contact endoscope (Karl Storz, Tuttlingen, Germany). Informed written consent was obtained from all patients, while the study protocol met the criteria of the Declaration of Helsinki in its latest version and was reviewed and approved by the local ethics committee (report no. 49/18). Patients with multiple laryngeal lesions or multiple biopsies were excluded. 

**Figure 1:**
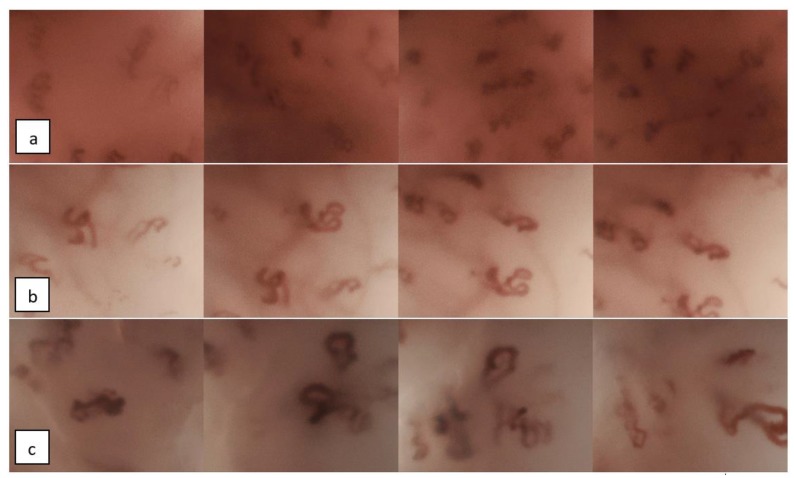
Examples of perpendicular vascular changes (PVC)-positive lesions: series of contact endoscopy with narrow-band imaging (NBI-CE) images depicting PVC. Histological diagnoses: (**a**) squamous cell carcinoma (SCC), (**b**) carcinoma in situ, and (**c**) papillomatosis.

Three otolaryngology specialists (experienced observers) and three otolaryngology residents (less-experienced observers), blinded to the histologic diagnoses and macroscopic image of every lesion, independently evaluated each of the 68 series of NBI-CE images, aiming to detect PVC. In cases where the observer detected at least one PVC in one or more of the images available, the lesion was characterized as PVC-positive and therefore suspect for malignancy, dysplasia, or papilloma (Figure 1a–c). 

**Figure 2:**
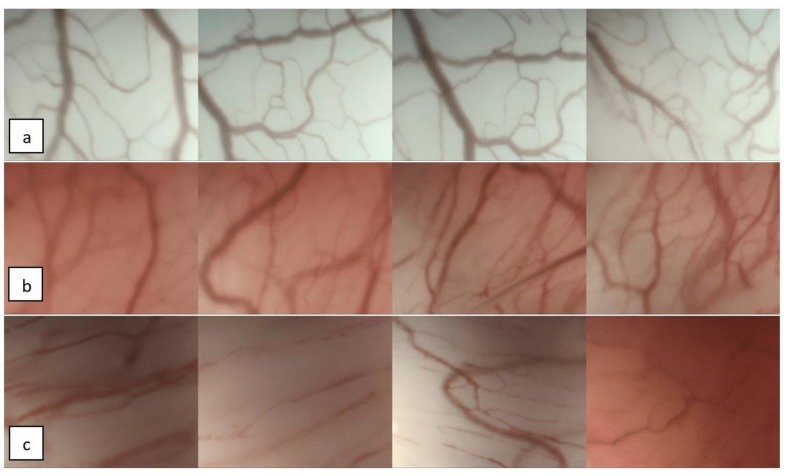
Examples of PVC-negative lesions: series of NBI-CE images depicting longitudinal vascular changes (LVC). Histological diagnoses: (**a**) Reinke’s edema, (**b**) cyst, and (**c**) nodule.

In all other cases, the lesions were declared PVC-negative (Figure 2a–c).

For the statistical assessment of the results, the histological diagnoses were grouped into four categories: 1. squamous cell carcinoma (SCC), 2. dysplasia (including mild dysplasia to carcinoma in situ according to the WHO classification of 2005 or low to high-grade dysplasia according to the WHO classification of 2017 [13]), 3. papillomatosis, and 4. other benign lesions.

The prevalence of reported PVC-positive lesions from the experienced and less-experienced observers in the four histological categories was determined. The number of PVC-positive lesions in relation to the groups of diagnoses were compared using the Chi-Square test for both groups of observers. PVC-positive lesions, with the histological diagnosis of SCC or dysplasia, were considered as true positives for the calculations of sensitivity, specificity, and positive and negative predictive values (PPV and NPV, respectively). Fleiss’ kappa (interpreted according to Landis and Koch [14]) was used to evaluate the interobserver agreement in the detection of PVC for the experienced and less-experienced observers. The statistical calculations were performed with IBM SPSS Statistics software package (version 26) and Microsoft Excel 2016. *P* values < 0.05 and nonoverlapping of 95% confidence intervals were considered statistically significant.

## 3. Results

### 3.1. Reported Prevalence of PVC-Positive Lesions in Different Groups of Histological Diagnoses

Sixty-eight patients having 68 histologically examined laryngeal lesions were included in the study. These encompassed eight lesions with the histological diagnosis of SCC, 17 with dysplasia, 11 with papillomatosis, and 32 with another benign vocal fold lesion. The latter covered the diagnoses of Reinke’s edema (12), polyps (5), hyperkeratosis (4), cysts (3), squamous hyperplasia (3), amyloidosis (2), fibroma (1), granuloma (1), and vocal fold nodule (1). The (averaged) proportions of reported PVC-positive lesions among the experienced and less-experienced observers ranged from 62.5%–87.5% for SCC, 70.6%–88.2% for dysplasia, 90.9%–100% for papillomatosis, and 15.6%–21.9% for the other benign lesions (Table 1).

### 3.2. Association of the Detection of PVC to Different Groups of Histological Diagnoses

Comparing the evaluations of the observers (Chi-Square test), the detection of PVC in NBI-CE images showed a negative association to benign lesions (other than papillomatosis) and a positive association to papillomatosis and dysplasia, for both experienced and less-experienced observers (*p* < 0.000). In addition to that, for experienced observers, the detection of PVC showed a positive association to SCC (*p* < 0.000). 

### 3.3. Sensitivity, Specificity, and Positive and Negative Predictive Values in the Diagnosis of Malignant and Premalignant Laryngeal Lesions

The sensitivity, specificity, and positive and negative predictive values in the diagnosis of dysplasia or SCC were 0.955, 0.630, 0.553, and 0.967, respectively, for the experienced observers and 0.727, 0.609, 0.471, and 0.824 for the less-experienced observers (Table 2). The sensitivity and negative predictive values were significantly higher in the experienced group.

### 3.4. Interobserver Agreement in the Identification of PVC in NBI-CE

The level of agreement between the observers in the identification of PVC, as depicted in the Fleiss’ kappa statistics, was moderate for the less-experienced group and almost perfect for the experienced group (Table 3). There was a significant difference between the calculated kappa values of the two groups. The overall interobserver agreement was substantial (kappa 0.703).

## 4. Discussion

### 4.1. Endoscopic Evaluation of Vascular Patterns of the Vocal Folds

The evaluation of superficial vascular patterns of the vocal folds has emerged in the last years as an important element in the diagnostic workup of vocal fold lesions, in addition to the evaluation of the mucosal surface or volume changes using conventional WLE and the evaluation of vibratory patterns using stroboscopy and high-speed imaging [15]. Modern image-enhancing technologies, such as NBI, SPIES, or i-SCAN, and the technical evolution of endoscopes, which nowadays enable a high image resolution up to 4K, allow a detailed description of minute mucosal and vascular changes [7]. As a result, nonspecific termini such as erythroplakia should be replaced through more precise descriptions of mucosal and vascular patterns [6], leading to increases in the accuracy and reproducibility of endoscopic diagnostics in preoperative and intraoperative settings [3,16,17,18]. NBI, designed to enhance the visibility of superficial vessels, is established as the primary image-enhancing modality for the evaluation of vascular changes of the vocal folds [7,11,19,20,21,22]. CE, introduced to laryngology through Andrea et al. in the 1990s, has been combined with image-enhancing modalities such as autofluorescence as early as 2003 [9,23]. Recently, CE has been combined with NBI or SPIES for visualizing vascular patterns in 60× (or 150×) magnification mode without any need for tissue staining [5,6]. The visualization of the magnified and highly contrasted vocal fold vessels enables a detailed in vivo evaluation of vascular patterns and tumor margins during laryngeal surgery [5,10].

### 4.2. ELS Classification for Vascular Changes of the Vocal Folds

Different classification systems for the vascular changes of vocal folds were introduced in the last years, while specific patterns have been associated with different histological diagnoses [4,5,6,24]. The proposal of the Committee on Endoscopic Laryngeal Imaging of the ELS for a descriptive guideline for vascular changes of the vocal folds introduced a simplified approach divided between longitudinal and perpendicular vascular changes. The latter represents intracapillary papillary capillary loops (IPCL) and develops in the context of neoangiogenesis and tumorigenesis as a result of viral or other exogenous factors. PVC have been associated with laryngeal papillomatosis, precancerous, and cancerous lesions [6]. This dichotomous distinction can serve in the endoscopic diagnosis of premalignant and malignant vocal fold lesions, as laryngeal papillomatosis can easily be distinguished due to its characteristic macroscopic appearance on conventional WLE or using NBI [7,12]. In addition to that, a further subdivision between wide and narrow-angled points in PVC can assist in the differentiation between (pre)malignant lesions and papillomatosis [6,8]. On the other hand, the detection of such patterns and the identification of PVC are partly subjective and can depend on the clinical experience and learning curve of the observer [5,16]. 

### 4.3. Key Findings of the Present Study in Relation to Previous Research

To our best knowledge, this is the first multiobserver study evaluating the presence of PVC as described in the ELS proposal using NBI-CE. The reported prevalence of PVC for experienced observers was 87.5%–88.2% for laryngeal dysplasia and SCC, 100% for papillomatosis, and 15.6% for other benign lesions. A clear association could be shown between the presence of PVC and SCC, dysplasia, and papillomatosis, as well as an association of the lack of PVC to benign lesions other than papillomatosis, as suggested by Arens et al. [6]. Furthermore, the interobserver agreement in the detection of PVC was almost perfect (Fleiss’ kappa 0.920) for experienced observers, suggesting no relevant variability in the evaluation of vascular patterns using the ELS classification. The achieved mean sensitivity of 0.955 and negative predictive value of 0.967 point to a high clinical value in the context of excluding malignancy. The mean specificity of 0.630 and positive predictive value of 0.533 mainly depict the classification of laryngeal papillomatosis (based on vascular patterns) to the neoplastic lesions, in concordance with the findings of previous studies evaluating vascular classification systems using NBI alone [8,16]. The evaluation of a subdivision of PVC according to the shape of their turning points as presented by Sifrer et al. was not applied in the present study [8].

For the less-experienced observers, the reported prevalence of PVC was 62.5%–70.6% for laryngeal dysplasia and SCC, 90.9% for papillomatosis, and 21.9% for other benign lesions. As in the experienced group, an association between PVC and the diagnoses dysplasia and papillomatosis, and an association between the lack of PVC and benign lesions (other than papillomatosis), could be confirmed. Furthermore, there was a moderate interobserver agreement in the detection of PVC (Fleiss’ kappa 0.510) suggesting that less-experienced observers can adequately use the proposed ELS classification. The agreement was lower than in the experienced group, similarly to other studies on interobserver agreement using NBI or WLE [25]. The mean sensitivity (0.727), specificity (0.609), and positive and negative predictive values (0.471 and 0.824, respectively) were, as expected, lower than in the experienced group. The differences for specificity and PPV were not significant. Without any doubt, in lack of relevant clinical experience, a considerable learning curve in the interpretation of endoscopic findings should be expected, as highlighted by other authors [2,5,11].

The comparability of the present work to previous studies in the field is low, as most of them used different endoscopic modalities, did not solely focus on vascular pattern changes, and applied other classification systems to categorize them [2,4,26]. The ELS classification system was proved reliable in recent studies on laryngeal lesions using image-enhancing techniques without CE [8,18,21]. Puxeddu et al. used CE, mainly in combination with SPIES in different modi (Clara + Chroma to Spectra A and B), for the endoscopic diagnoses of laryngeal and hypopharyngeal lesions. The authors of the study could prove a progressive irregularity in vascular patterns moving from inflammation and hyperplasia to dysplasia and invasive cancer and proposed a new classification system [5]. To date, there is sparse literature on the comparison of different classification systems of vascular changes of the vocal folds. There is some evidence that the use of the ELS classification in diagnosing malignancy is preferential to that proposed by Ni et al. from the clinical perspective, as it has been characterized as easier to use, with comparable accuracy [7]. The reproducibility of the evaluation using the ELS system could be confirmed from our data, both for experienced and less-experienced observers.

Concluding, the intraoperative use of NBI-CE for the detection and clinical interpretation of vascular changes of the vocal folds proved to be a valuable tool for both experienced and less-experienced observers. The classification proposed by the ELS can be used in combination with NBI-CE for the differentiation between (pre)malignant lesions (and papillomatosis) and benign lesions (other than papillomatosis), allowing experienced surgeons to optimize therapeutic plans and avoid unnecessary multiple surgeries. A combination of NBI-CE to other preoperative or intraoperative endoscopic modalities can probably further increase the diagnostic efficacy of the method, as proposed in previous studies [18,27]. For daily clinical practice, it seems reasonable to propose a detailed intraoperative examination of specific regions of interest using NBI-CE following the examination of any suspect lesion with WLE and an image-enhancing modality such as NBI. 

### 4.4. Implications and Suggestions for Future Research

The evaluation of larger patient groups in multicentral studies in the future could provide more information about the value of NBI-CE in different clinical situations. The present data indicates the need for further research in diagnostic methods based on the endoscopic evaluation of vascular patterns. A comparison of different classification systems could provide more evidence on their advantages and disadvantages in diagnosing malignancy. Furthermore, as in other medical fields like gastroenterology, such endoscopic data can be used for automated evaluation approaches [28]. Training machine-learning algorithms to differentiate between benign and malignant lesions, as proposed by Esmaeili et al., could play an important role in computer-aided diagnoses in the future [29]. Automated vascular pattern recognition and assessments could be used alone or in combination with texture assessment strategies to optimize endoscopic diagnoses in the future [30,31]. Further research in that field is needed to prove its value in everyday clinical practice.

## 5. Conclusions

The use of NBI-CE is a reliable endoscopic modality for the evaluation of vascular changes of the vocal folds. The proposed ELS classification can help experienced and less-experienced observers differentiate between (pre)malignant lesions (and papillomatosis) and benign lesions (other than papillomatosis). 

## Figures and Tables

**Table 1:** Proportions (values averaged over observers) of reported PVC-positive lesions in different groups of histological diagnoses, according to the evaluations of experienced and less-experienced observers.

Histological Diagnosis	No. of Lesions	Experienced Observers	Less-Experienced Observers
SCC ^1^	8	87.5%	62.5%
Dysplasia	17	88.2%	70.6%
Papillomatosis	11	100.0%	90.9%
Other benign lesions	32	15.6%	21.9%

^1^ SCC: Squamous Cell Carcinoma.

**Table 2:** Sensitivity, specificity, and positive and negative predictive values, according to the evaluation of experienced and less-experienced observers (values averaged over observers with 95% confidence intervals).

	Experienced Observers		Less-Experienced Observers	
		95%–CI ^1^		95%–CI ^1^
Sensitivity	0.955	0.905–1.004	0.727	0.621–0.833
Specificity	0.630	0.516–0.745	0.609	0.493–0.725
PPV ^2^	0.553	0.434–0.671	0.471	0.352–0.589
NPV ^3^	0.967	0.924–1.009	0.824	0.733–0.914

^1^ CI: confidence interval, ^2^ PPV: positive predictive value, and ^3^ NPV: negative predictive value.

**Table 3:** Interobserver agreement concerning the identification of PVC-positive lesions.

Observers	Fleiss’ Kappa	95%–CI ^1^	Agreement According to [14]
Experienced	0.920	0.783–1.058	almost perfect
Less Experienced	0.510	0.373–0.647	moderate
Overall	0.703	0.642–0.764	substantial

^1^ CI: Confidence interval.
